# Combining bulk and scRNA‐seq to explore the molecular mechanisms governing the distinct efferocytosis activities of a macrophage subpopulation in PDAC

**DOI:** 10.1111/jcmm.18266

**Published:** 2024-03-19

**Authors:** Shaoliang Zhu, Quan Cheng, Mengjie Zou, Chunxing Li, Yi Tang, Longjie Xia, Yanming Jiang, Zheng Gong, Zhenyong Tang, Yuntian Tang, Honglin Luo, Ningfu Peng, Xiaojing Wang, Xiaofeng Dong

**Affiliations:** ^1^ Department of Hepatobiliary, Pancreas and Spleen Surgery The People's Hospital of Guangxi Zhuang Autonomous Region, Guangxi Academy of Medical Sciences Nanning China; ^2^ Department of General Surgery The First Affiliated Hospital of Nanjing Medical University Nanjing China; ^3^ Department of Nephrology The People's Hospital of Guangxi Zhuang Autonomous Region, Guangxi Academy of Medical Sciences Nanning China; ^4^ Department of Operating Room The People's Hospital of Guangxi Zhuang Autonomous Region, Guangxi Academy of Medical Sciences Nanning China; ^5^ Department of Cosmetology and Plastic Surgery Center The People's Hospital of Guangxi Zhuang Autonomous Region, Guangxi Academy of Medical Sciences Nanning China; ^6^ Department of Gynecology The People's Hospital of Guangxi Zhuang Autonomous Region, Guangxi Academy of Medical Sciences Nanning China; ^7^ Department of Anesthesiology The People's Hospital of Guangxi Zhuang Autonomous Region, Guangxi Academy of Medical Sciences Nanning China; ^8^ Institute of Oncology, The People's Hospital of Guangxi Zhuang Autonomous Region, Guangxi Academy of Medical Sciences Nanning China; ^9^ Department of Hepatobiliary Surgery Guangxi Medical University Cancer Hospital Nanning China; ^10^ Department of Rheumatology and Immunology, Tongren Hospital, School of Medicine Shanghai Jiao Tong University Shanghai China

**Keywords:** cellular signalling network, efferocytosis, immunotherapy, macrophage, single‐cell

## Abstract

Pancreatic ductal adenocarcinoma (PDAC), a very aggressive tumour, is currently the third leading cause of cancer‐related deaths. Unfortunately, many patients face the issue of inoperability at the diagnostic phase leading to a quite dismal prognosis. The onset of metastatic processes has a crucial role in the elevated mortality rates linked to PDAC. Individuals with metastatic advances receive only palliative therapy and have a grim prognosis. It is essential to carefully analyse the intricacies of the metastatic process to enhance the prognosis for individuals with PDAC. Malignancy development is greatly impacted by the process of macrophage efferocytosis. Our current knowledge about the complete range of macrophage efferocytosis activities in PDAC and their intricate interactions with tumour cells is still restricted. This work aims to resolve communication gaps and pinpoint the essential transcription factor that is vital in the immunological response of macrophage populations. We analysed eight PDAC tissue samples sourced from the gene expression omnibus. We utilized several software packages such as Seurat, DoubletFinder, Harmony, Pi, GSVA, CellChat and Monocle from R software together with pySCENIC from Python, to analyse the single‐cell RNA sequencing (scRNA‐seq) data collected from the PDAC samples. This study involved the analysis of a comprehensive sample of 22,124 cells, which were classified into distinct cell types. These cell types encompassed endothelial and epithelial cells, PDAC cells, as well as various immune cells, including CD4+ T cells, CD8+ T cells, NK cells, B cells, plasma cells, mast cells, monocytes, DC cells and different subtypes of macrophages, namely C0 macrophage TGM2+, C1 macrophage PFN1+, C2 macrophage GAS6+ and C3 macrophage APOC3+. The differentiation between tumour cells and epithelial cells was achieved by the implementation of CopyKat analysis, resulting in the detection and categorization of 1941 PDAC cells. The amplification/deletion patterns observed in PDAC cells on many chromosomes differ significantly from those observed in epithelial cells. The study of Pseudotime Trajectories demonstrated that the C0 macrophage subtype expressing TGM2+ had the lowest level of differentiation. Additionally, the examination of gene set scores related to efferocytosis suggested that this subtype displayed higher activity during the efferocytosis process compared to other subtypes. The most active transcription factors for each macrophage subtype were identified as BACH1, NFE2, TEAD4 and ARID3A. In conclusion, the examination of human PDAC tissue samples using immunofluorescence analysis demonstrated the co‐localization of CD68 and CD11b within regions exhibiting the presence of keratin (KRT) and alpha‐smooth muscle actin (α‐SMA). This observation implies a spatial association between macrophages, fibroblasts, and epithelial cells. There is variation in the expression of efferocytosis‐associated genes between C0 macrophage TGM2+ and other macrophage cell types. This observation implies that the diversity of macrophage cells might potentially influence the metastatic advancement of PDAC. Moreover, the central transcription factor of different macrophage subtypes offers a promising opportunity for targeted immunotherapy in the treatment of PDAC.

## 1 INTRODUCTION

Pancreatic ductal adenocarcinoma (PDAC) is a malignancy characterized by its high degree of aggressiveness, hence presenting a substantial menace to the overall well‐being of the population. Despite the progress made in the field of medicine, there has been a consistent increase in the occurrence and fatality rates of PDAC at an average yearly rate of 0.3%. This upward trend may be attributed mostly to shifts in lifestyle patterns, the aging demographic and extended life spans. At now, it holds the position as the third most prominent cause of cancer‐related mortalities, with projections indicating its ascent to the second position by the year 2030.^1^ The identification of the disease during its initial phases presents a notable obstacle because of the lack of discernible symptoms or biological markers. Furthermore, the illness exhibits a quick progression, and the availability of surgical intervention is frequently limited at diagnosis. Moreover, traditional therapeutic interventions have demonstrated little efficacy in managing the neoplasm, leading to a modest 5‐year survival rate of about 10%.^2^ Overall, PDAC has evolved into a malignancy that poses a serious hazard to human health and is receiving increasing attention.

According to research, the complex interplay between PDAC cells and their environment can have a major influence on tumour development, progression and resistance. Experts in this field have committed their efforts to unravelling the intricate mechanisms that underpin these events in order to obtain a better understanding of them.^3^, ^4^, ^5^ Despite extensive and diligent endeavours, the outcomes have been unsatisfactory. The 5‐year survival rate for PDAC is comparatively lower than that of other kinds of tumours. The primary reason for this is the inherent constraints of traditional research approaches when investigating tumour heterogeneity. The cellular signalling network is a multifaceted system that encompasses a diverse array of signalling molecules and receptors, functioning inside the confines of the cell as well as between cells. It regulates cellular communications, encompassing both internal and external interactions. The origin of cancer is closely associated with the complicated network present inside cells.

Single‐cell sequencing is an advanced approach that allows for the sequencing of genetic material from individual cells, enabling the analysis of their genome, transcriptome, epigenetic changes and other relevant biological information.^6^, ^7^, ^8^, ^9^, ^10^ The process involves several consecutive stages: The experimental approach includes isolating individual cells from recently acquired tissue samples and then breaking down the cells to liberate their contents. The nucleic acids are collected from the lysed cells, followed by a sequence amplification procedure. This amplified material is then subjected to sequencing and the resulting data is analysed.^11^ Single‐cell sequencing has made great progress and is widely used in scientific and therapeutic fields. Diagnosing and treating PDAC is challenging because to the intricate interaction of several variables and the notable diversity in the tumour microenvironment. Recent progress in single‐cell sequencing technology now allows for detailed analysis of genomic, transcriptomic or epigenomic characteristics of individual cells. This skill has shown to be very advantageous in revealing previously undiscovered molecular pathways that are the basis of PDAC. Consequently, the use of single‐cell sequencing has become a pivotal instrument in the realm of PDAC investigation. The phenomenon has catalysed substantial advancements in our understanding of the ailment and the formulation of innovative therapeutic approaches.^12^, ^13^, ^14^

Metastatic progression, which refers to the dissemination of cancer cells to distant anatomical sites, constitutes a prominent etiological factor contributing to mortality in individuals diagnosed with PDAC. Regrettably, those who get metastases typically receive palliative treatment and encounter a bleak prognosis. Despite early detection and surgical treatment, a majority of patients are likely to encounter a relapse within a span of 4 years, indicating the possible existence of early micrometastases in PDAC.^2^ To enhance the outlook for persons with PDAC, it is crucial to fully comprehend the mechanisms behind metastasis and create efficient strategies for preventing and treating metastatic disease. PDAC is known for its quick progress. The specific mechanisms driving the evolution of PDAC are still not fully understood, even though several genetic variations linked to tumour development have been identified by genomic and transcriptome studies. Therefore, creating successful treatments presents a substantial therapeutic obstacle. The diversity of PDAC is a substantial challenge for researchers due to differences seen among tumour forms and within certain microenvironments. Single‐cell sequencing technology is now a potent tool for studying the genetic and functional variations within malignancies and for pinpointing unique cell subgroups. This method shows potential for enhancing our comprehension of tumour development. Recent research has demonstrated that cancers can exploit efferocytosis, a kind of cell death, to advance cancer growth and evade immune system detection. However, the level of efferocytosis activity exhibited by macrophages in PDAC remains uncertain.^15^, ^16^

The proposed study aims to employ single‐cell sequencing technology to examine the efferocytosis activity, transcriptional regulatory networks and cellular communication mechanisms between macrophages and tumour cells in the setting of PDAC. This methodology will facilitate our comprehension of the molecular and cellular connections that underlie this particular ailment, perhaps yielding significant insights into innovative therapy options.

## 2 METHODS

### 2.1 Sample acquisition

The single‐cell RNA sequencing (scRNA‐seq) data for PDAC were acquired from the gene expression omnibus (GEO) database under the accession number GSE156405 (https://www.ncbi.nih.gov/geo/). The dataset consisted of eight samples. External validation group single cell data from GSE197177. The Seurat software version 4.1.1 was employed to import 10X genomics data from each sample into R software version 4.1.3. Initially, the DoubletFinder (v2.0.3) software was employed to identify any instances of doublets arising from cell encapsulation or fortuitous pairings of cells and samples that were not adequately segregated throughout the process of sample generation. In this investigation, cellular entities that exhibited an expression level above 20% of the aggregate count of expressed genes, namely mitochondrial genes, were excluded. The exclusion criteria were the removal of cells that exhibited suboptimal quality, characterized by a gene count of less than 200, a gene count above 6000, and a cell count below three. As the data utilized in this investigation was obtained from a publicly accessible database, it was not necessary to get ethical approval.

As the data utilized in this investigation was obtained from a publicly accessible database, ethical approval was not deemed necessary.

### 2.2 Identification of cell clusters

The log(x+1) method was employed to determine the level of gene expression in each cells, represented as a fraction of the gene multiplied by 10,000, in order to do the natural logarithm transformation. The expression matrix was utilized for the purpose of identifying the 2000 genes exhibiting the highest degree of variability, commonly referred to as highly variable genes (HVGs). Subsequently, the genes underwent a principal component analysis (PCA) to facilitate scaling. The R Harmony package (version 1.0) was employed to mitigate batch effects by utilizing the top 50 PCA components. The computation of k‐nearest neighbours was performed using data that has been adjusted for harmony. Subsequently, a graph known as the shared nearest neighbour (SNN) graph was constructed. The clustering technique subsequently modified the modular function in order to get cluster identification. The clusters that were identified were shown on a two‐dimensional map created utilizing the uniform manifold approximation and projection (UMAP) technique for reducing dimensionality.

The marker genes for each cluster were identified using the ‘FindAllMarkers’ function, employing the following parameters: logfc.threshold = 0.25, min.pct = 0.25, and min.diff.pct = 0.25. The utilization of the DotPlot tool led to the further use of the Seurat DotPlot and featureplot tools in order to visually represent the expression patterns of individual marker genes across different clusters. The categorization of cell groups was performed by utilizing differentially expressed genes (DEGs) and established cellular markers that have been documented in the scientific literature. In order to better investigate the heterogeneity of PDCA cells, a process of re‐clustering was conducted on the PDCA cells. Subsequently, the identification of each cluster of PDCA cells was performed based on their distinct genetic profiles.

### 2.3 Single cell copy number variation (CNV) evaluation

The CNV level was computed using Copykat (version 1.1.0) and inferCNV (version 1.12.0). Copy‐number karyotyping of aneuploid tumours was developed using copy‐kat to distinguish between malignant and non‐malignant cell types. InferCNV was used to determine whether or not additional cancer cells show significant chromosomal CNV by using the NK cell as a reference.

### 2.4 Cell–cell communication between PDAC cells and subtypes of macrophage cells

Based on RNA sequencing data from a single cell, the CellChat tool (version 1.4.0) indicated that cell types could communicate. A *p*‐value of 0.05 was used to cell–cell interactions to predict cell–cell contact in different cell types.

### 2.5 Pseudotemporal ordering of macrophage cells

The Monocle programs (version 2.22.0) was utilized to investigate the pseudo‐time trajectories of different kinds of macrophage cells. The objective of monocle is to elucidate the biochemical alterations occurring throughout the process of macrophage cell differentiation. The ‘newCellDataSet’ function was utilized to incorporate the scale of raw UMI counts and its clustering information. Subsequently, the data was transformed into a reduced dimensional space utilizing the discriminative dimensionality reduction with trees (DDRTree) approach, which is a contemporary technique for manifold learning.

### 2.6 Gene set variation analysis (GSVA), single‐sample gene set enrichment analysis (ssGSEA), gene set enrichment analysis (GSEA), kyoto encyclopedia of genes and genomes (KEGG) and gene ontology (GO) analysis among macrophage cells subtypes

In order to determine the functional significance of the DEGs, an analysis was conducted on the upregulated genes using the GO database (http://www.geneontology.org/).^17^ The identification of GO concepts with varying levels of enrichment was performed by the use of hypergeometric testing. The likelihood‐ratio test was employed to identify genes exhibiting differential expression across many macrophage cell types. The *p*‐values in this study were subjected to adjustment in order to accommodate the false discovery rate (FDR), and a *p*‐value of 0.05 was identified as possessing statistical significance. The analysis employed GSEA software version 4.1.0 to investigate gene function. The MSIGDB database from the GSEA website (http://software.broadinstitute.org/gsea/msigdb) was applied for this purpose.^18^, ^19^, ^20^ In order to identify the pathways that exhibit the greatest differences across subgroups, we employed the technique of differential gene induction to rank them. The Pi package (version 2.6.0) and MsigdbH were utilized to conduct a gene set enrichment analysis. The majority of study in the field has been concentrated on the 50 signature pathways that are included in the molecular signature database. Furthermore, we evaluated the efficacy of efferocytosis by examining data from macrophage cells. Subsequently, the GSVA program (version 1.42.0) was employed to allocate route activity estimates to individual cells. GSVA and ssGSEA are novel methodologies utilized for the identification of active genes in scRNA‐seq data. The input for GSVA or ssGSEA consists of a predefined set of genes, referred to as a gene set. The result of these analyses is the quantification of the ‘activity’ of the gene set inside each individual cell.

### 2.7 Scenic analysis of macrophage cells subtypes

By utilizing scRNA‐seq data, the SCENIC method is able to rebuild gene regulatory networks and effectively identify cell states that exhibit stability. This work utilized Python (version 3.7) and the pySCENIC package (version 0.10.0) to assess the enrichment of transcription factors and the activity of regulons. The development of the gene regulatory network was based on co‐expression and DNA motif analysis. The determination of the cell state was accomplished by the analysis of network activity inside each individual cell. The gene‐motif ranking within a 10 kb region around the transcription start site was employed as a basis for determining the search area for transcription factor regulatory networks in proximity to the transcription start site. The motif rankings for human genes are obtained from the website https://resources.aertslab.org/cistarget/.

### 2.8 Immunofluorescence of PDAC tissues

To explore the presence of efferocytosis mediated by macrophages in PDAC. According to the manufacturer's instructions, Multicolor IHC staining was performed using a Multiple Immunofluorescence Kit (AiFang biological, AFIHC035). Briefly, the paraffin‐embedded tissues were dewaxed, and then the antigen retrieval was performed by sodium citrate at 98°C for 20 min. After infiltrating in 3% H_2_O_2_ for 15 min, the tissues were blocked by 5% BSA. Then, the tissues were incubated by primary antibody at 4°C overnight. Then, the tissues were incubated by secondary antibody for 30 min and stained by TSA for 5 min. For each target, the steps of antigen retrieval, blocking, incubation of primary antibody, incubation of secondary antibody, and TSA staining are required. Finally, the nuclei were stained with DAPI. PBS was used to wash tissues between each step. These images were obtained by the LEICA DMi8 system and analysed by the ImageJ software.

### 2.9 The prognostic implications of macrophage subtypes

According to the GDC TCGA database, the construction of predictive gene signatures involved utilizing genes that are associated with specific cell subtypes of interest. The PDAC validation set data is obtained from four different datasets: GSE62452, GSE71729, GSE78229 and GSE85916. The objective of this study was to investigate the contribution of certain cell subtypes in predicting patient survival. The study first utilized a univariate Cox regression analysis to investigate the specific cell subtypes of interest in relation to marker genes, specifically focusing on the top 100 genes. The present investigation has successfully discovered genes that have a statistically significant correlation with patient prognosis (*p* < 0.05). To address the problem of multicollinearity among the genes, we performed further screening on these genes using LASSO regression (glmnet, version 4.1–6).^21^, ^22^, ^23^, ^24^, ^25^ The risk coefficients for each gene were subsequently estimated using multivariate Cox regression analysis. The study incorporated survival analyses using the Kaplan–Meier technique, which was executed using the R package Survival (version 3.3–1). Furthermore, the ggsurvplot program was employed to graphically depict the survival curves. Following this, a time‐varying receiver operating characteristic (ROC) curve was produced to assess the predictive capacity of the model.^26^, ^27^, ^28^ The study was performed with the time ROC R package (version 0.4). In addition, a multivariate Cox regression analysis was performed in order to evaluate the statistical significance of risk gene and cell type scores as autonomous prognostic factors.

### 2.10 The assessment of the immunological microenvironment

The immunological microenvironment was evaluated using the CIBERSORT R package (version 0.1.0) method. The study encompassed the assessment of 22 distinct immune cell types present in the tumour immunological microenvironment of various patient cohorts, followed by the computation of scores corresponding to each individual immune cell type. The objective of this study was to assess and contrast the extent of immune cell infiltration in the two cohorts. In addition, we conducted an evaluation of the tumour microenvironment by utilizing the ‘ESTIMATE,’ ‘CIBERSHOT’ and ‘Xcell’ algorithms. Subsequently, we merged the obtained scores to comprehensively analyse the tumour microenvironment. The concept of the microenvironment pertains to the local vicinity and circumstances that exert a direct impact on an organism or system. The computation of the immune microenvironment score, comprising the stromal score and immunological score, was performed using the ESTIMATE R package (version 1.0.13). The scores obtained were subsequently shown using the reshape2 R package (version 1.4.4) and ggpubr R package (version 0.6.0). Spearman's correlation analysis was employed to examine the relationship between the risk score and the genes that comprise the risk score. In addition, the tumour immune dysfunction and exclusion (TIDE) algorithm, accessible at http://tide.dfci.harvard.edu/, was utilized to assess the efficacy of immune checkpoint inhibitors (ICIs) in the treatment of cancer patients.

## 3 RESULTS

### 3.1 Quality control

DoubletFinder (version 2.0.3) was used to identify and eliminate doublets prior to additional investigation. Cells that expressed more than 20% of all the genes expressed as mitochondrial genes were therefore disregarded from our study. Notably, cells with more than 6000 genes, less than three cells, or low‐quality cells with fewer than 200 genes were eliminated. Batch effects were reduced using Harmony (version 0.1.0) using the top 30 PCA components among samples.

### 3.2 Cell annotation

**FIGURE 1:**
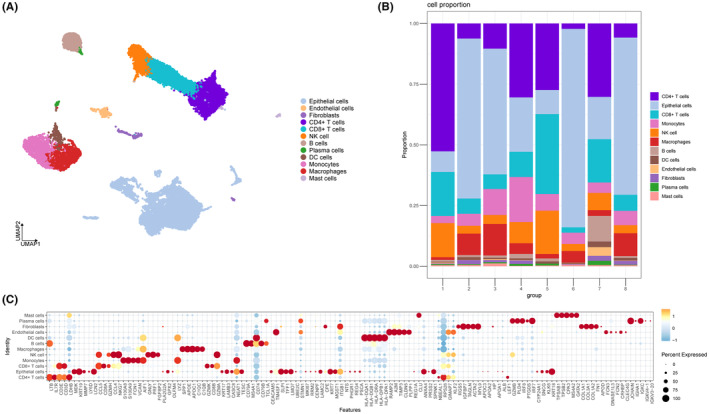
Identification of the cell types. (A) The 2D plots of UMAP dimensionality reduction of cell type. (B) The percentage of cell types between samples was represented on a proportion chart. (C) Dot plot showing top5 marker genes for cell types.

After batch effect correction, 22,124 cells were categorized into 27 clusters at a resolution of 1.0. To decrease dimensionality, we utilized UMAP methods, and the outcome was shown as a 2D scatter diagram (Figure 1A). The sample cluster percentages were shown in the proportion chart (Figure 1B). To show the expression of specific markers inside clusters, a dot plot was used (Figure 1C).

We labelled the names of these clusters as follows: Monocytes (cluster 3), NK cells (cluster 4), Macrophages (cluster 6), B cells (cluster 9), DC cells (cluster 14), Endothelial cells (clusters 15, 25), Fibroblasts (clusters 18, 24), Plasma cells (clusters 21, 26), Mast cells (cluster 23). These clusters were identified based on the specific expression genes of 27 clusters.

### 3.3 Complex heterogeneity within epithelial cells

**FIGURE 2:**
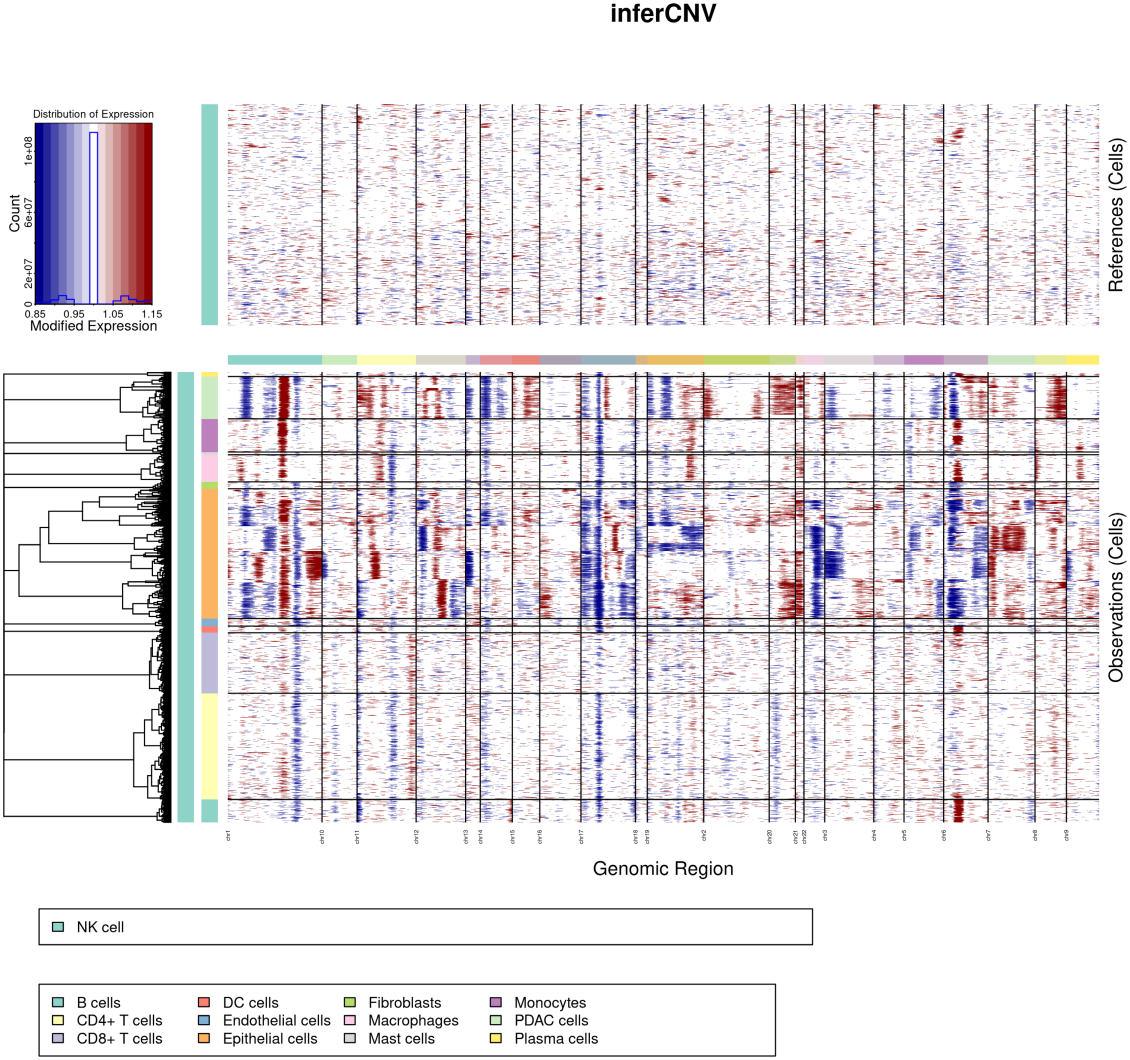
Heatmap of inferred copy number variation (CNV) across all cells. The heatmap displayed large‐scale CNVs of PDAC cells and epithelial cells. The red colour represents a high CNV level and the blue represents a low CNV level.

CopyKat analysis was performed to identify the tumour cells in epithelial cells. A total of 1941 PDAC cells were identified from epithelial cells (Figure S1). InferCNV analysis was used to infer the CNV status of cells from different cell types using NK cells as controls (Figure 2). Similar CNV scores were obtained in PDAC cells and other epithelial cells, indicating tumour‐like cells in epithelial cells. Besides, PDAC cells were characterized by their unique CNV amplification/deletion on different chromosomes compared with epithelial cells.

### 3.4 Identification of macrophage cell subtypes

**FIGURE 3:**
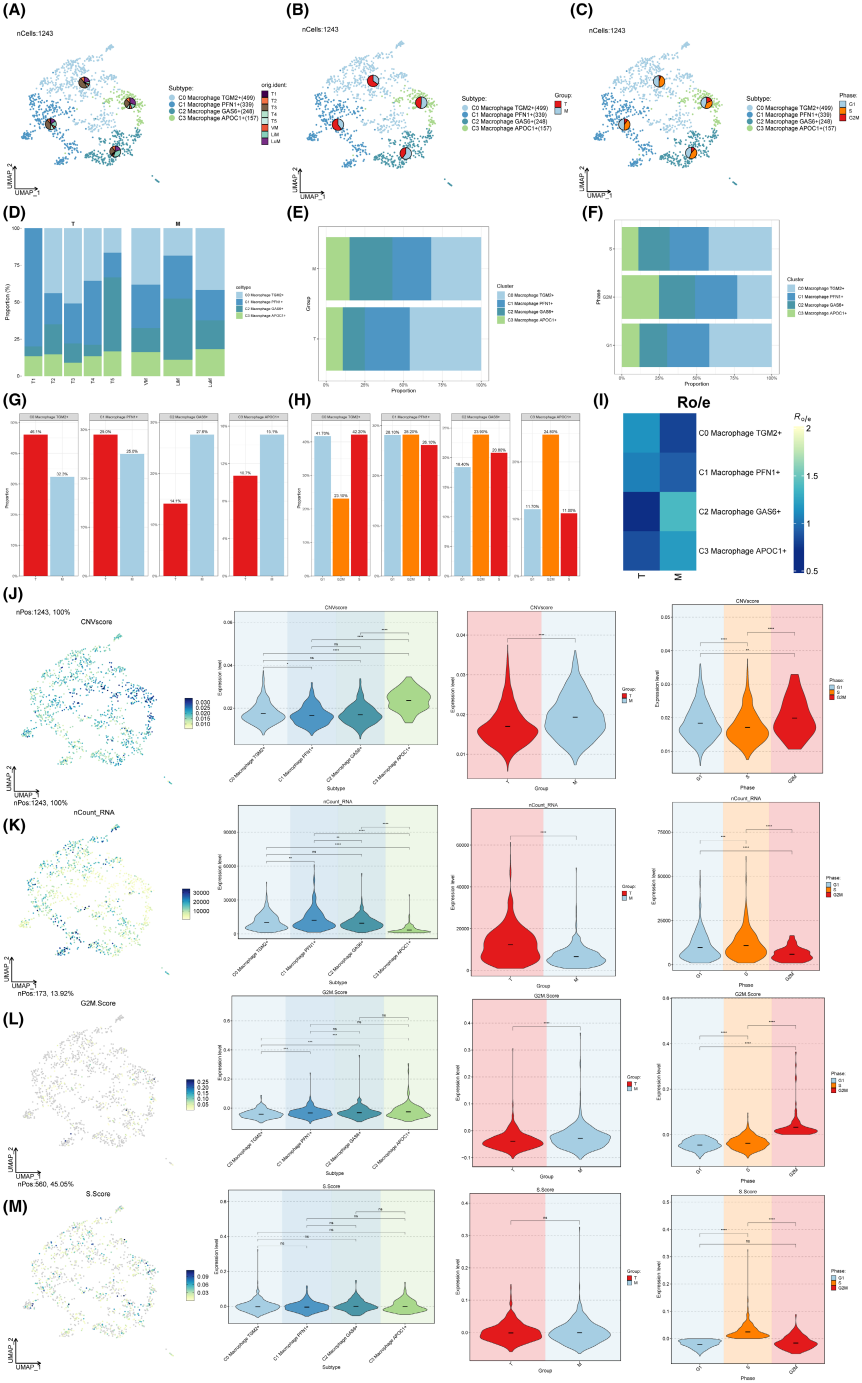
Subtypes of macrophage cells. (A–C) The 2D plots of UMAP dimensionality reduction of subtypes of macrophage cells. (D–H) Bar graph illustrated the distribution of macrophage cell subtypes among several groups. (I) The ratio of observed to predicted cell counts (Ro/e) was computed for each cluster across several tissues in order to assess the tissue preference of each cell subtype. (J–M) CNV score, nCount, G2M.Score and S. Score were shown by UMAP plots and violin plots between groups.

A total of 1243 macrophage cells were clustered into four subtypes (C0 Macrophage TGM2+, C1 Macrophage PFN1+, C2 Macrophage GAS6+, C3 Macrophage APOC1+) (Figure 3A–C). The bar charts depicted the percentages of macrophage cell subtypes between different groups (Figure 3D–H). We calculated the ratio of observed to expected cell numbers (Ro/e) for each cluster in different tissues to quantify the tissue preference of each cell subtype (Figure 3I). UMAP plots, and violin plots of CNV score, nCount, G2M.Score, and S.Score among macrophage cell subtypes were shown in Figure 3J–M. The myeloid cells of GSE197177 dataset that have been grouped into 16 distinct cell clusters. (Figure S2A).

### 3.5 Communication networks between macrophage cell subtypes and PDAC cells

**FIGURE 4:**
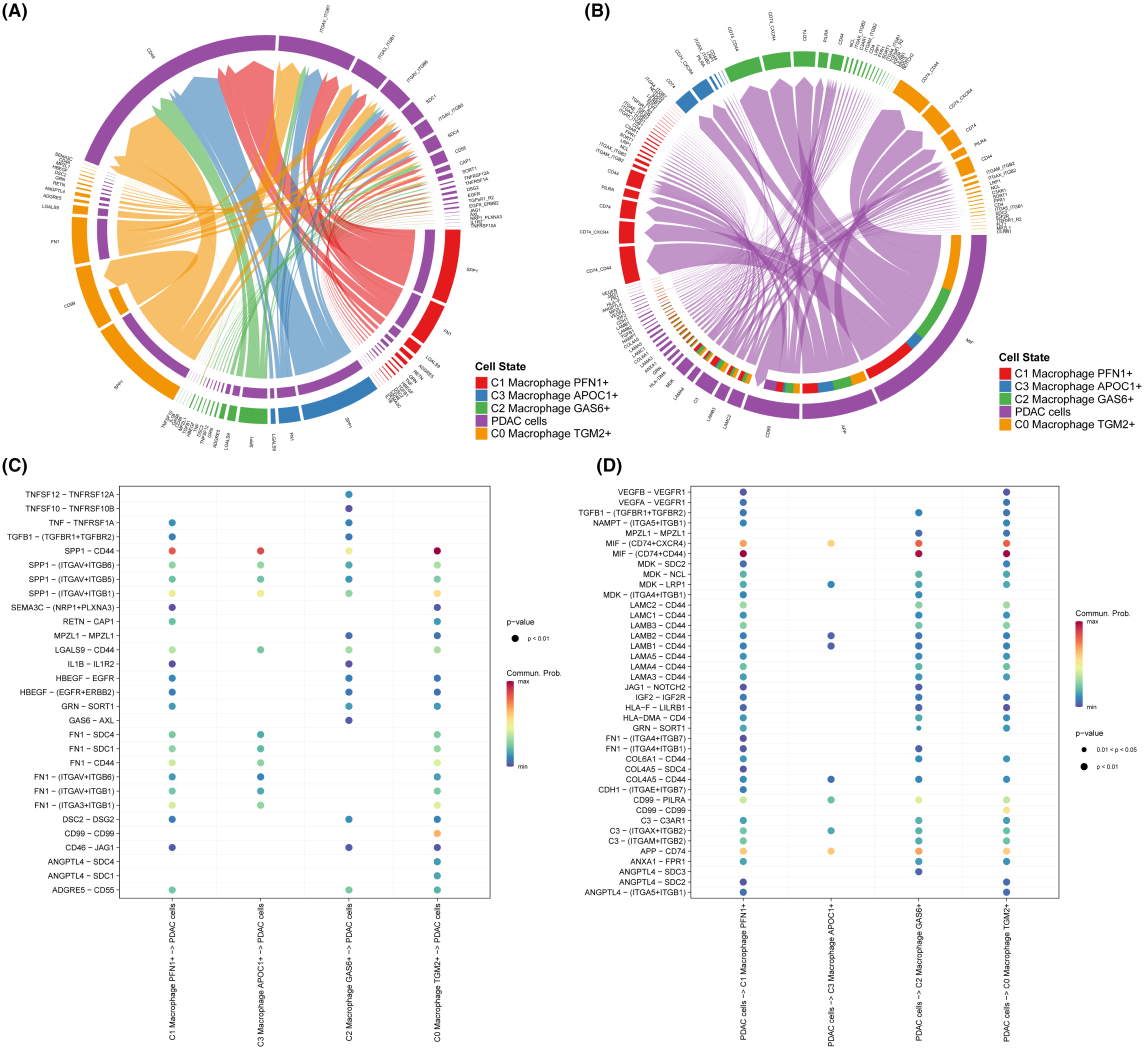
CellChat analysis between PDAC cells and subtypes of macrophage cells. (A) Chordal graph of the overall of ligand–receptor pairs between subtypes of macrophage cells and PDAC cells. (B) Chordal graph of the overall of ligand–receptor pairs between PDAC cells and subtypes of macrophage cells. (C) Dot plot of the overall of ligand–receptor pairs between subtypes of macrophage cells and PDAC cells. (D) Dot plot of the overall of ligand–receptor pairs between PDAC cells and subtypes of macrophage cells.

The CellChat program (version 1.4.0) was used to investigate possible interactions between macrophage cell subtypes and PDAC cells. Chordal graphs and dot plots were used to illustrate the overall potential for communication between macrophage cell subtypes and PDAC cells (Figure 4). From the above results, we can concluded that the potential communication between macrophage cell subtypes and PDAC cells mainly thorough SPP1‐CD44, SPP1‐(ITGAV+ITGB1), and CD99‐CD99 signalling network. Therefore, the potential communication between PDAC cells and macrophage cell subtypes mainly involved in MIF‐(CD74 + CXCR4), MIF‐(CD74 + CD44) and APP‐CD74 signalling network.

### 3.6 Pseudotime trajectories analysis of macrophage cell subtypes

**FIGURE 5:**
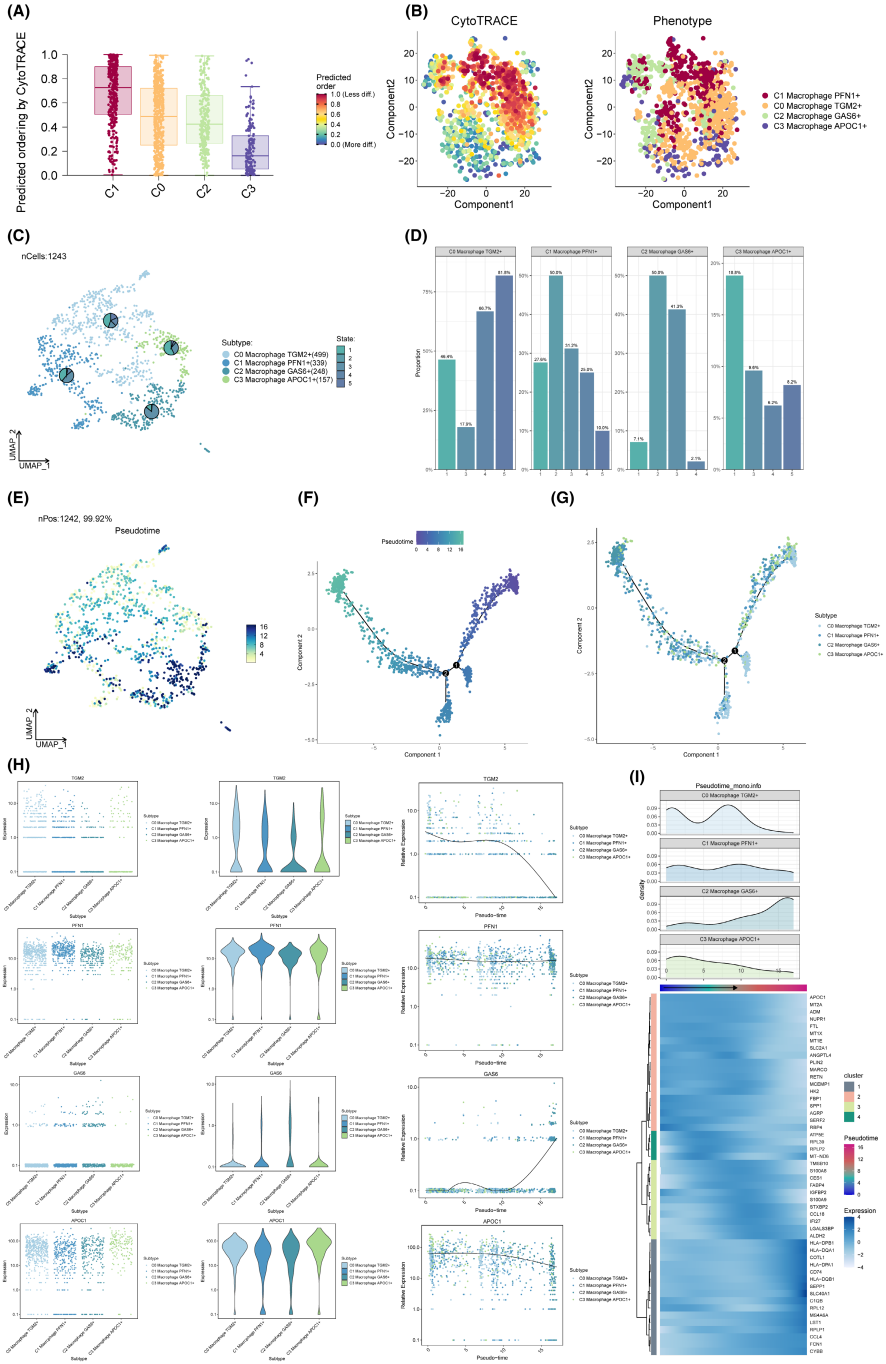
Pseudotime trajectories analysis of subtypes of macrophage cells. (A, B) The CytoTRACE was utilized to examine cell stemness among, macrophage cell subtypes to investigate the aspects of macrophage cell subtype differentiation. (C–G) The results of Monocle show that C0 macrophage TGM2+ has the lowest degree of differentiation compared to other macrophage cell subtypes. (H) The changes in expression of macrophage cell subtype markers (APOC1, GAS6, PFN1, and TGM2) as the pseudotime trajectory continued. (H) Heatmaps based on pseudotime trajectory analysis were used to provide the top indicators for each macrophage cell subtype.

To explore the features of macrophage cell subtype differentiation, the CytoTRACE was used to analyse the cell stemness among macrophage cell subtypes. The results showed that, compared to other subtypes, C0 macrophage TGM2+ and C1 macrophage PFN1+ have higher cellular stemness (Figure 5A,B). In addition, the Monocle (version 2.22.0) was used to analyse macrophage cell subtype differentiation. Compared to other macrophage cell subtypes, C0 macrophage TGM2+ has the lowest degree of differentiation, as determined by the findings of the Monocle assay (Figure 5C–G). As the pseudotime trajectory progressed, the expression alterations of the markers for the subtypes of macrophage cells (APOC1, GAS6, PFN1 and TGM2) were shown in Figure 5H. In addition, the top markers for each macrophage cell subtype were presented in heatmaps based on pseudotime trajectory analysis (Figure 5I).

### 3.7 Efferocytosis gene set scoring and enrichment analysis among macrophage cells subtypes

**FIGURE 6:**
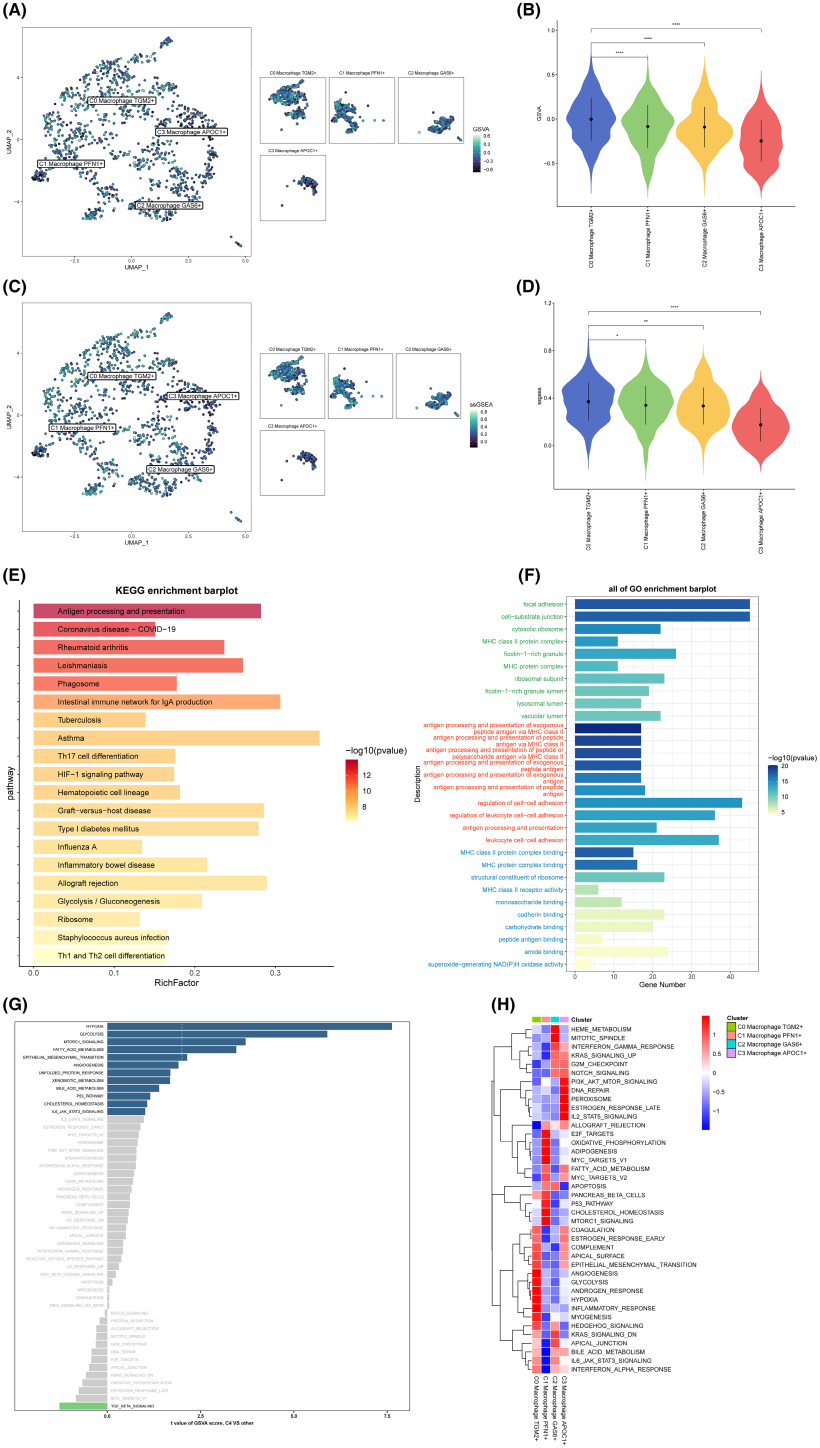
GSVA, ssGSEA, GSEA, KEGG, and GO analysis among macrophage cells subtypes. (A, B) Differences in efferocytosis related genes activities scored per cell by GSVA between macrophage cells subtypes. (C, D) Differences in efferocytosis related genes activities scored per cell by ssGSEA between macrophage cells subtypes. (E) KEGG enrichment analysis between C4 Macrophage CES1+ and other macrophage cell subtypes. (F) GO enrichment analysis between C4 Macrophage CES1+ and other macrophage cell subtypes. (G) GSVA enrichment analysis between C4 Macrophage CES1+ and other macrophage cell subtypes. (H) GSEA enrichment analysis between C4 Macrophage CES1+ and other macrophage cell subtypes.

The efferocytosis gene set scoring results revealed that C0 macrophage TGM2+ has more activity in the efferocytosis process than other subtypes (Figure 6A–D). Therefore, the KEGG (Figure 6E), GO (Figure 6F), GSVA (Figure 6G) and GSEA (Figure 6H) enrichment analyses of DEGs between C0 macrophage TGM2+ and other macrophage cell subtypes were subsequently used to explore the pathway activity difference. The results of KEGG showed that the DEGs mainly enrich in Antigen processing and presentation, Coronavirus disease—COVID‐19, Rheumatoid arthritis, Leishmaniasis, Phagosome, Intestinal immune network for IgA production, Tuberculosis, Asthma, Th17 cell differentiation, HIF − 1 signalling pathway, Haematopoietic cell lineage, Graft−versus−host disease, Type I diabetes mellitus, Influenza A, Inflammatory bowel disease, Allograft rejection, Glycolysis/Gluconeogenesis, Ribosome, Staphylococcus aureus infection and Th1 and Th2 cell differentiation. The results of GO showed that the DEGs mainly enrich in superoxide−generating NAD(P)H oxidase activity, amide binding, peptide antigen binding, carbohydrate binding, cadherin binding, monosaccharide binding, MHC class II receptor activity, structural constituent of ribosome, MHC protein complex binding, MHC class II protein complex binding, leukocyte cell–cell adhesion, antigen processing and presentation, regulation of leukocyte cell–cell adhesion, regulation of cell–cell adhesion, antigen processing and presentation of peptide antigen, antigen processing and presentation of exogenous antigen, antigen processing and presentation of exogenous peptide antigen, antigen processing and presentation of peptide or polysaccharide antigen via MHC class II, antigen processing and presentation of peptide antigen via MHC class II, antigen processing and presentation of exogenous peptide antigen via MHC class II, vacuolar lumen, lysosomal lumen, ficolin−1 − rich granule lumen, ribosomal subunit, MHC protein complex, ficolin−1 − rich granule, MHC class II protein complex, cytosolic ribosome, cell−substrate junction and focal adhesion. In addition, the results of GSVA enrichment analysis indicated that the activity of HYPOXIA, GLYCOLYSIS, MTORC1_SIGNALLING, FATTY_ACID_METABOLISM, EPITHELIAL_MESENCHYMAL_TRANSITION, ANGIOGENESIS, UNFOLDED_PROTEIN_RESPONSE, XENOBIOTIC_METABOLISM, BILE_ACID_METABOLISM, P53_PATHWAY, CHOLESTEROL_HOMEOSTASIS, IL6_JAK_STAT3_SIGNALLING in C0 macrophage TGM2+ higher than other subtypes. Moreover, the results of GSEA enrichment analysis also showed the difference in pathway activity between C0 macrophage TGM2+ and different subtypes.

### 3.8 Scenic analysis of macrophage cells subtypes

**FIGURE 7:**
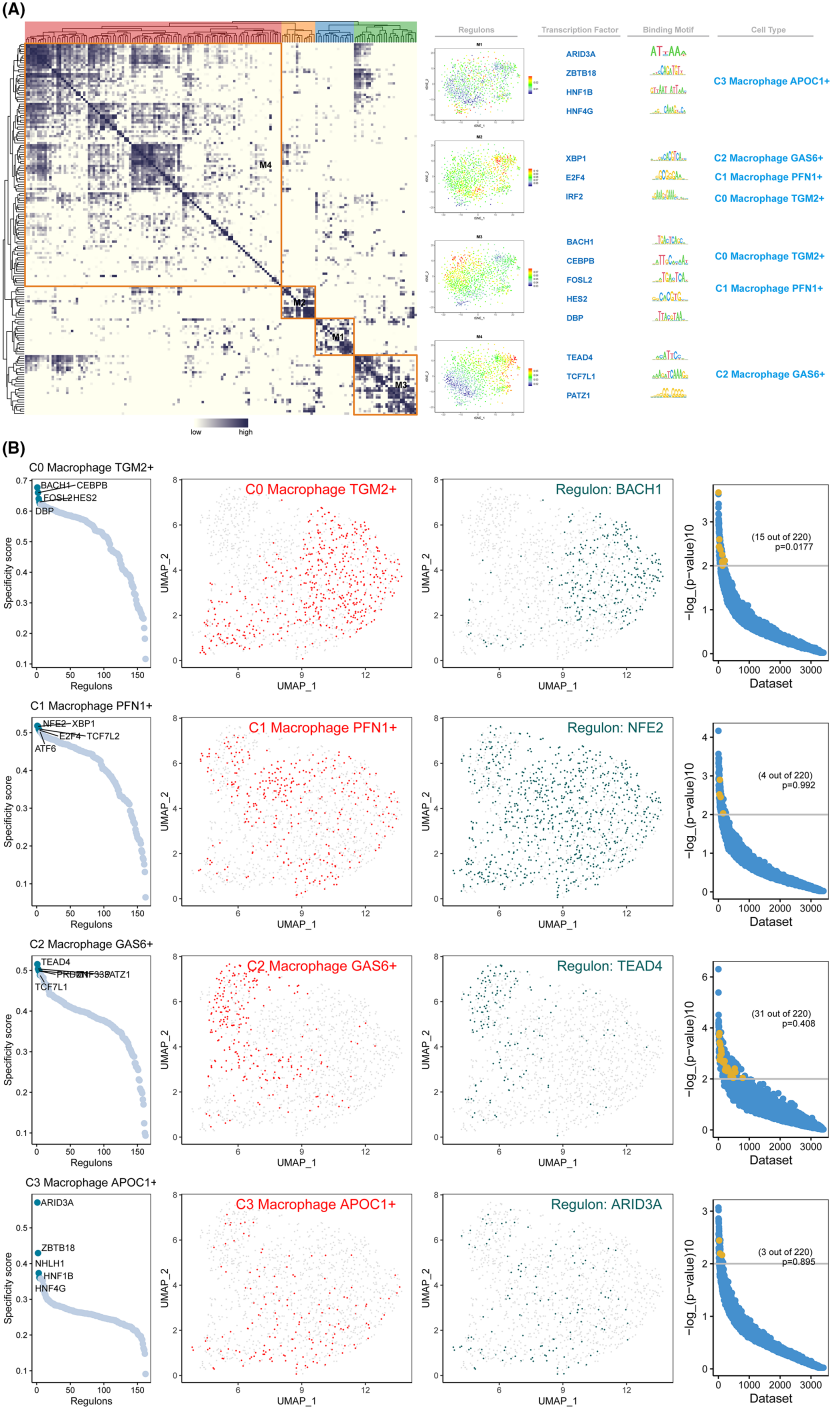
Gene regulatory network analysis of macrophage cell subtypes. (A) Based on the connection specificity index (CSI) matrix, regulon modules of macrophage cell subtypes, representative transcription factors, related binding motifs, and connected macrophage cell subtypes were identified. (B) Rank for regulons in macrophage cell subtypes based on regulon specificity score (RSS). Macrophage cell subtypes are highlighted in the t‐SNE (red dots). Binarized regulon activity scores (RAS) for the top regulon of macrophage cell subtypes on t‐SNE (perform Z score normalization across all samples, and set 2.5 as the cutoff to convert to 0 and 1) (dark green dots). SEEK was used for co‐expression results for the top CEBPD regulon target genes. The x‐axis indicates the various datasets, while the y‐axis indicates the significance of co‐expression of target genes in each dataset.

A SCENIC analysis was conducted to find the core TFs identifiable in subclusters of macrophage cell subtypes. We utilized pySCENIC to reconstruct the gene regulatory networks of all of the subclusters of PDCA, and we categorized these regulons into four primary modules (M1, M2, M3 and M4). The usual TFs and cell types were determined based on the average activity ratings of each module. ARID3A, ZBTB18, HNF1B and HNF4G, which are important regulators for C3 macrophage APOC1+, were located in Module M1. The regulators XBP1, E2F4 and IRF2 in Module M2 were related to C2 macrophage GAS6+, C1 macrophage PFN1+ and C0 macrophage TGM2+. Module M3 consisted of BACH1, CEBPB, FOSL2, HES2 and DBP, in addition to regulators for C0 macrophage TGM2+ and C1 macrophage PFN1+. Module M4 comprises regulators, including TEAD4, TCF7L1 and PATZ1, related to the C2 macrophage GAS6+ (Figure 7A).

In addition, the UMAP plots give additional evidence that the actions of these TFs are highly unique to the respective subtypes of macrophage cells. When we mapped the average activity score of each module into UMAP, we discovered that each module occupies a special zone, and all highlighted areas display complimentary patterns. According to the results of the study on cell type‐specific regulon activity, BACH1 (C0 macrophage TGM2+), NFE2 (C1 macrophage PFN1+), TEAD4 (C2 macrophage GAS6+) and ARID3A (C3 macrophage APOC1+) are the most active transcription factors of these macrophage cell subtypes (Figure 7B).

### 3.9 Immunofluorescence

**FIGURE 8:**
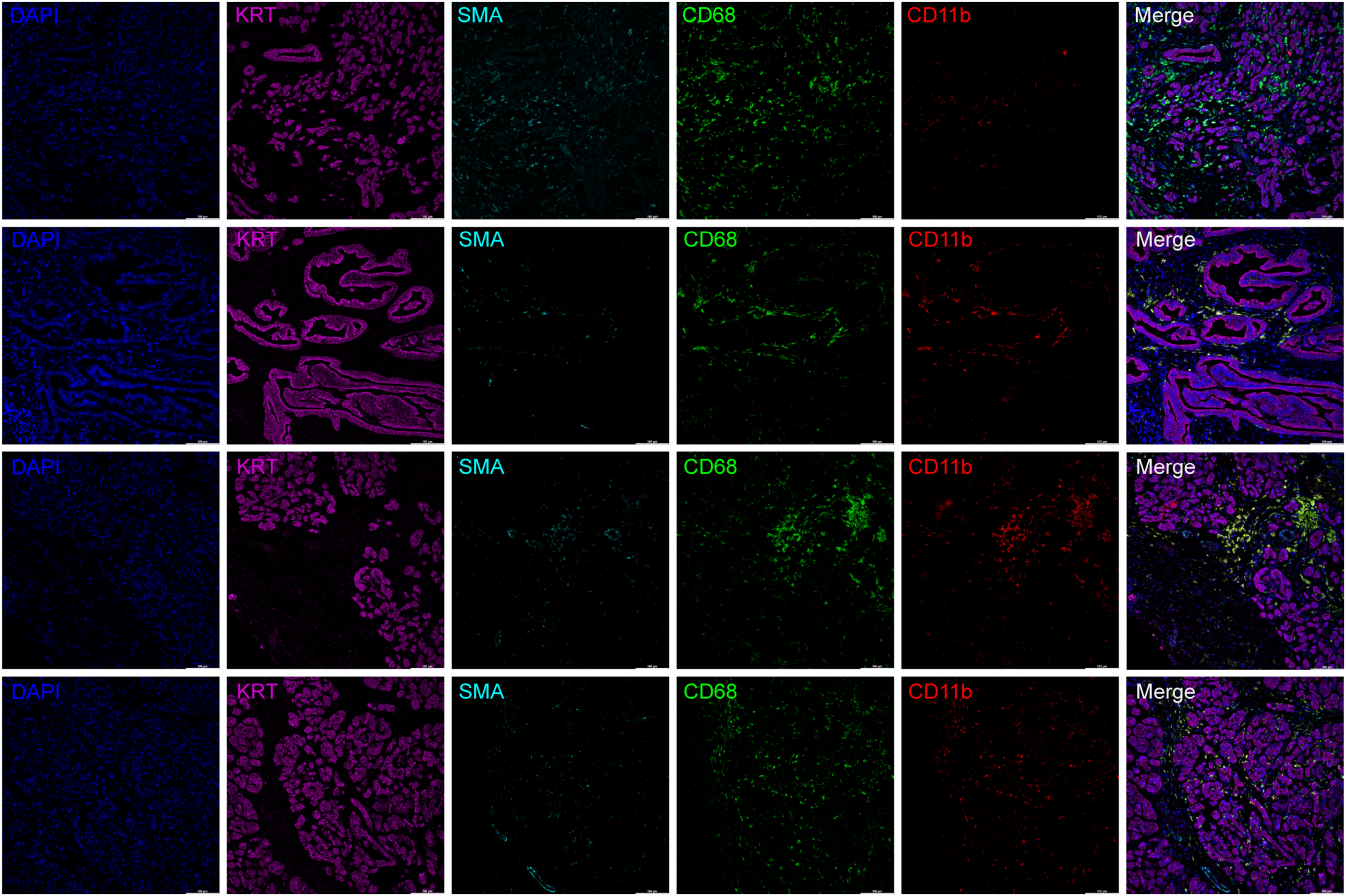
Multicolor IHC staining. Multicolor IHC staining with anti‐KRT (purples), anti‐SMA (cyan), anti‐CD68 (green), anti‐CD11b (red) and DAPI (blue) was exemplified by PDAC patients (*n* = 4). The green and red indicate CD68 + CD11b + macrophage cells. The scale bar represents 100 μm.

To explore the spatial location among macrophages, fibroblasts, and epithelial cells in PDAC, we performed immunofluorescence in human PDAC tissues, staining CD11b with CD68, a classical macrophage marker. Results showed spatial co‐localization between CD68 and CD11b in KRT‐ and α‐SMA‐ regions (Figure 8).

### 3.10 The AUCell score of C0 Macrophage TGM2 Top100 in the other PDAC myeloid subgroup

The AUCell algorithm was used to score other PDAC myeloid cell subpopulations based on the C0 Macrophage TGM2 Top100 genes list. The results indicated that the C0 Macrophages, C1 Macrophages, C5 Macrophages, C6 Macrophages, C13 Macrophages and C14 Macrophages had a higher score, implying the presence of a subpopulation resembling the C0 Macrophage TGM2 in the macrophages of this PDAC dataset (Figure **S2**B,C).

### 3.11 Risk score associated with C0 macrophage TGM2+

**FIGURE 9:**
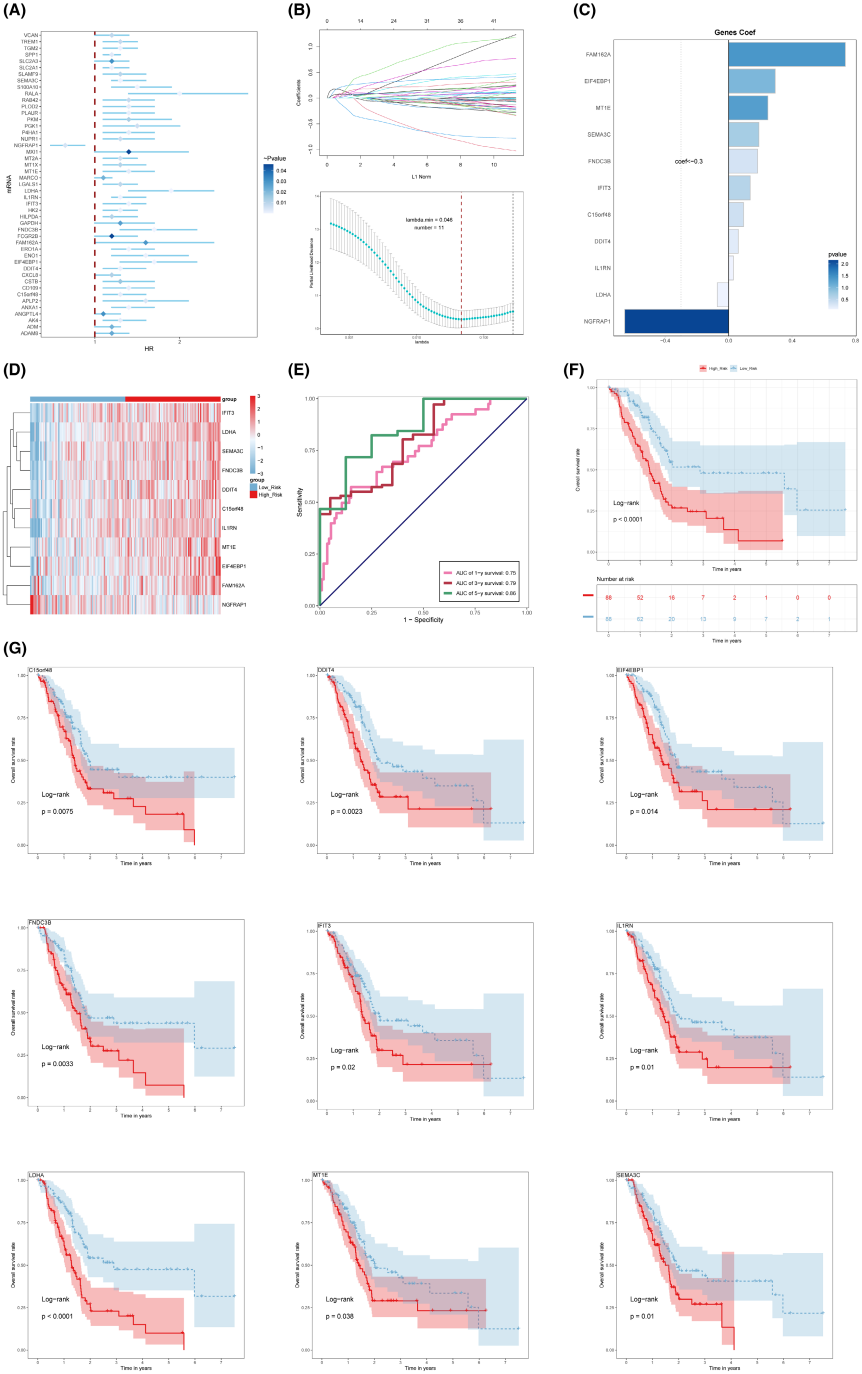
Risk score associated with C0 macrophage TGM2+. (A) Forest graphs depicted the results of a Univariate Cox analysis of the top 100 differential genes in C0 macrophage TGM2+. (B) By employing LASSO (Least Absolute Shrinkage and Selection Operator) regression analysis, 11 genes associated with prognosis were identified. (C) A bar graph displaying the Coef (coefficient) value of the genes constituting risk score. (D) Heatmap demonstrated the expression of these genes between groups. (E) The results of ROC curves for risk score (AUC values for 1 year, 3 years, and 5 years). (F) The Kaplan–Meier curve showed a difference in survival between the High score group (high_risk) and the Low score group (low_risk). (G) The Kaplan–Meier analysis of genes constituting risk score revealed a statistically different prognosis between High expression group (represented by the colour red) and the Low expression group (represented by the colour blue).

To enhance the prediction accuracy of the risk profile, we developed a risk scoring system based on top 100 markers of C0 macrophage TGM2+. Initially, an examination was conducted on these genes by the utilization of Univariate Cox regression. Subsequently, genes that exhibited an association with patient prognosis were chosen based on a significance level of *p* < 0.05, as depicted in Figure 9A. To mitigate the issue of multicollinearity among the genes, we conducted further screening of these genes with LASSO regression. The results of this analysis revealed a total of 11 genes that exhibited a significant association with the prognosis of patients, as seen in Figure 9B. The risk scores in our study incorporated a total of 11 genes, and the coefficients for these genes were determined using Multivariate Cox regression analysis, as seen in Figure 9C. Furthermore, we computed the relevant risk coefficients based on the individual risk coefficients of each gene. Risk score = expression level of LDHA *‐0.071229981 + expression level of C15orf48 * 0.094439554 + expression level of DDIT4 * 0.061394024 + expression level of SEMA3C * 0.191018369 + expression level of MT1E * 0.247419683 + expression level of FAM162A * 0.735819898+ expression level of EIF4EBP1 * 0.293977082+ expression level of IL1RN * 0.030124271+ expression level of NGFRAP1 *‐0.654896642+ expression level of FNDC3B * 0.182860888+ expression level of IFIT3 * 0.136203193. Based on the formula, we conducted risk score calculations for each patient and then classified them into two groups: the High score group (high_risk) and the Low score group (low_risk), with the median value serving as the cutoff point. Additionally, heat maps are utilized to illustrate the differential expression of genes constituting risk score in the two groups (Figure 9D). To evaluate the prognostic efficacy of the risk score, the area under the ROC curves was employed to ascertain the precision in forecasting the outcomes at the first, third and fifth years within the TCGA cohort. The findings indicated that the area under the ROC curve (AUC) values were 0.75, 0.79 and 0.86 for the first year, third year and fifth year, respectively (Figure 9E). When examining the differences in survival rates between the High score group and the Low score group, it was seen that the Low score group had more favourable survival results compared to the High score group (Figure 9F). The survival analysis was conducted to examine the differential expression of the following genes: C15orf48, DDIT4, EIF4EBP1, FNDC3B, IFIT3, IL1RN, LDHA, MT1E, SEMA3C. The analysis compared the High expression group (represented by the colour red) with the Low expression group (represented by the colour blue), using the median expression value as the cutoff criterion. It was discovered that the survival outcomes for these genes that were had a higher level of superiority in the Low expression group as opposed to the High expression group (Figure 9G). Furthermore, we conducted external validation of this risk score using the GSE62452, GSE71729, GSE78229 and GSE85916 datasets. The survival curves demonstrated that patients with PDAC in the High_Risk group had a significantly poorer prognosis, aligning with the findings from the initial modelling dataset (Figure S3).

### 3.12 Analysis of immune infiltration, tumour mutational burden (TMB) between high score group (high_risk) and the low score group (low_risk)

**FIGURE 10:**
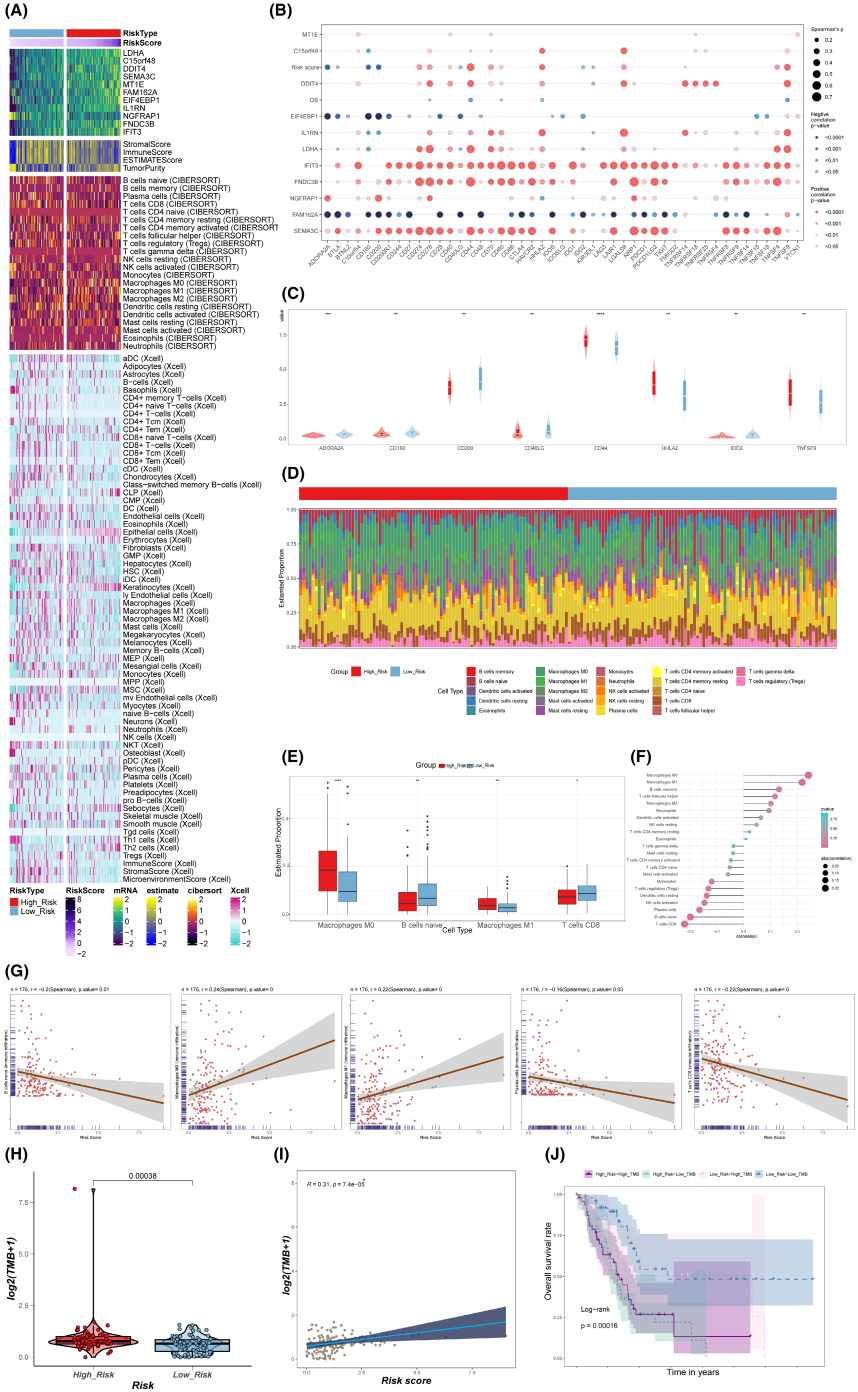
The analysis of ESTIMATE, CIBERSORT, Xcell, SNP, and TMB. (A) Heatmap showed the results of ESTIMATE, CIBERSORT, Xcell in PDAC. (B) Bubble chart showed the correlation analysis between immune checkpoint associated genes and genes comprising. (C) The expression of immune checkpoint linked genes differed between the high score group (high_risk) and the LOW score group (low_risk). (D) The Cibersort analysis of the High score (high_risk) and low score (low_risk) groups. (E) The results of tumour‐infiltrating immune cells observed for PDAC. (F, G) The correlation analysis between immune infiltrating cells and the risk score. (H–J) The results of TMB between the low score group (low_risk) and the high score group (high_risk).

To obtain a thorough and cohesive understanding of the degree of immune infiltration, we conducted an analysis on the risk score within two groups: the High score group (high_risk) and the Low score group (low_risk). This analysis was performed using the ESTIMATE, Cibersort and xcell algorithms, as well as the ESTIMATEScore and cibersort metrics (Figure 10A).

Subsequently, we performed a correlation analysis to assess the relationship between immune checkpoint associated genes and genes comprising (Figure 10B). The expression differential of immune checkpoint associated genes between the High score group (high_risk) and the low score group (low_risk) was also investigated (Figure 10C).

To investigate the changes in immune cell composition between the High score group (high_risk) and the low score group (low_risk), we utilized the Cibersort analysis to explore these variations (Figure 10D). As seen in Figure 10E, the results revealed that there were greater proportion of tumour‐infiltrating immune cells observed for M0 and M1 in high score group (high_risk). The association between immune infiltrating cells and the risk score was also assessed, as seen in Figure 10F,G.

The TMB between the low score group (low_risk) and the high score group (high_risk) was then examined. TMB was greater in the high score group than in the low score group (*p* = 0.00038) (Figure **10**H). Figure **10**I displayed the association between TMB and risk score the high TMB and low TMB groups, as well as the high‐risk high mutation group, high‐risk low mutation group, low‐risk high mutation group, and low‐risk low mutation group, underwent survival difference analysis the findings are displayed in Figure **10**J.

## 4 DISCUSSION

Patients diagnosed with PDAC exhibit a notably poor 5‐year survival rate despite recent advances in treatment options.^29^ The development of new treatments and strategies for cancer is of utmost importance. Efferocytosis, a process that involves the clearance of apoptotic cells, has been the subject of extensive research in cancer.^16^ Efferocytosis is a physiological process that involves the removal of apoptotic cells. Macrophages and immature dendritic cells primarily induce this process and is crucial in regulating the inflammatory response and immune environment and promoting inflammation resolution and wound healing. Efferocytosis plays a vital role in maintaining tissue homeostasis and preventing the development of autoimmune diseases. Understanding the mechanisms behind this process is of great interest to researchers in immunology and cell biology.^30^ Macrophages are known to produce pro‐inflammatory cytokines such as type I interferon and interleukin‐12 in response to the consumption of pathogens. These cytokines serve as triggers for cytotoxic immune responses aimed at eradicating the invaders and any infected host cells.^31^ Furthermore, macrophages possess the remarkable capability to recognize and engulf their deceased cells. This initiates a cascade of signals that diminish pro‐inflammatory cytokines while augmenting the generation of anti‐inflammatory cytokines such as IL‐10 and wound‐healing cytokines like transforming growth factor (TGF)‐β. In the presence of these antigens displayed by macrophages, adaptive immune system T and B lymphocytes are signalled to tolerate the antigen and any cells that exhibit it due to the effects of anti‐inflammatory cytokines.^32^ In the clearance process, Tumour‐Associated Macrophages (TAMs) tend to exhibit a pro‐tumour M2‐like trait. This trait results in the production of anti‐inflammatory cytokines while suppressing the production of pro‐inflammatory cytokines. Consequently, immune selection occurs, leading to the clearance of apoptotic cells and the resolution of inflammation and immunosuppression. However, this favourable environment also enables cancer cells to evade immune surveillance, ultimately leading to tumour growth.^33^

Tumour‐associated macrophages, or TAMs, have been the subject of extensive research across various cancer types. TAMs have been shown to significantly promote tumour development, invasion, angiogenesis, and metastasis and are often associated with unfavourable prognoses for patients.^34^ According to a study by Ma et al., the SIAH2‐NRF1 pathway, including nuclear respiratory factor 1, influences the polarization of TAMs and cell death in breast cancer cells, ultimately promoting tumour maintenance through TME remodelling. The study also revealed that significant cell death can trigger cytokines like IL‐4, IL‐10, IL‐13, and TGF‐β, which aid in efferocytosis‐induced wound healing and further support the progression of metastatic tumours.^35^ According to a study by Jones and colleagues, M2 monocytes and macrophages have been shown to play a role in the spread of prostate cancer to the bones. This is due to their inherent ability to remove dead cells, creating a favourable tumour growth environment. However, trabectedin has been found to effectively target these phagocytic cells, significantly reducing the number of M2 macrophages. This ultimately leads to a decrease in the tumour size and an improvement in the bone microenvironment.^36^ Recent research demonstrating macrophage involvement even at the early stages of acinar cell dedifferentiation to ductal cells or acinar‐to‐ductal metaplasia highlights these cells' critical role in pancreatic cancer initiation, progression, and metastasis.^37^ Therefore, it is necessary to investigate further the mechanism of efferocytosis of macrophages in the TME of PDAC.

This study analysed single‐cell sequencing data from PDAC to investigate the diversity of macrophages and their signalling pathways with cancer cells. The study also aimed to identify key transcription factors involved in macrophage efferocytosis. After quality control, de‐batching effects, and initial annotation, 1243 macrophages were clustered into four subtypes (C0 Macrophage TGM2+, C1 Macrophage PFN1+, C2 Macrophage GAS6+, C3 Macrophage APOC1+). The results showed that PDCA macrophages exhibit high heterogeneity, with C0 macrophage TGM2+ being the least differentiated subtype. Additionally, C0 macrophage TGM2+ has more activity in the efferocytosis process than other subtypes. DEGs were analysed using KEGG, GO, GSVA, and GSEA to explore pathway activity differences. Results showed DEGs mainly enrich in antigen processing, peptide antigen binding, and MHC class II receptor activity. GSVA analysis revealed higher activity of HYPOXIA, GLYCOLYSIS, and MTORC1 signalling. The aforementioned findings may indicate that macrophages exhibiting vigorous efferocytosis possess elevated levels of HYPOXIA, GLYCOLYSIS.

Evidence that macrophage and cancer cells interact continuously in pancreatic cancer progression is gradually increasing.^38^, ^39^, ^40^ One study found that PDAC cells reprogrammed macrophages from M1‐like macrophages to M2‐like macrophages, followed by increased secretion of the cytokine IL‐10, which interacted with receptors on the surface of tumour cells and activated IL‐10/IL‐10R‐downstream signalling in tumour cells, promoting their metastasis.^41^ Yin et al. discovered that exosome miR‐501‐3p from M2 macrophages suppresses the tumour suppressor TGFBR3 gene and encourages the development of PDAC by activating the TGF‐signalling pathway.^42^ In experimental PDAC models, CSF1/CSF1R signalling in pancreatic tumours depletes CD206Hi TAMs and reprograms remaining macrophages to support anti‐tumour immunity.^43^ According to our research, there is a significant amount of communication taking place between cancer cells and macrophages. This was observed through single‐cell analysis, and we used CellChat analysis to identify specific potential pathways for communication between subtypes of macrophage cells and PDAC cells. We found that SPP1‐CD44, SPP1‐(ITGAV + ITGB1), and CD99‐CD99 ligand‐receptor pairs are potential pathways for communication between these cells. Additionally, we discovered that MIF‐(CD74 + CXCR4), MIF‐(CD74 + CD44) and APP‐CD74 ligand‐receptor pairs are likely pathways for communication between macrophage cell subtypes and PDAC cells. Our findings offer valuable evidence that a communication pathways may exist between pancreatic cancer cells and macrophages.

To further analyse key transcription factors associated with macrophages in PDCA, we used pySCENIC to reconstruct the gene regulatory network of all subclusters of PDCA. We grouped these regulators into four main modules (M1, M2, M3, and M4). The specific transcription factors corresponding to each subcluster, according to regulon specificity score results, were BACH1 (C0 macrophages TGM2+), NFE2 (C1 macrophages PFN1+), TEAD4 (C2 macrophages GAS6+) and ARID3A (C3 macrophages APOC1+). Several transcription factors have been identified as potentially valuable targets for cancer therapy. BACH1, for example, is highly expressed in tumours and is involved in epigenetic silencing during tumour progression.^44^ Inhibition of BACH1 has been shown to decrease antioxidant‐induced glycolysis rates and reduce migration and invasion of cancer cells.^45^, ^46^ Similarly, NFE2 and TEAD4 are also implicated in various types of cancers and are strongly associated with clinical significance. The transcription factor NFE2 commonly affects myeloproliferative tumours^47^ and has also been shown to be strongly expressed in endogenic mouse breast cancer cells. NFE2 can confer growth advantages on breast cancer cells under hypoxia and anchorage‐independent conditions,^48^ while TEAD4 expression is the most frequently observed among the four TEAD members in various types of cancers. According to a Pan‐Cancer investigation of entire genomes, 6% of the 2565 cancer patients analysed had TEAD4 abnormalities.^49^ ARID3A has been shown to regulate critical processes such as B lymphocyte maturation, macrophage maturation, and differentiation.^50^ The Knockdown of ARID3A could promote M1‐type polarization and inhibit M2‐type polarization, thus inhibiting tumour cell proliferation and metastasis.^51^ However, the reliability of these biomarkers needs to be validated with large amounts of clinical data and reliable cohort studies.

Our research has brought to light remarkable revelations about the varied characteristics of macrophages in PDAC. Our discoveries have led us to uncover previously undiscovered macrophage subtypes that strongly correlate with elevated efferocytosis. Additionally, we have pinpointed significant transcription factors closely tied to macrophages. These thrilling findings present new opportunities for exploring the underlying mechanisms of PDAC and developing personalized treatment solutions.

## AUTHOR CONTRIBUTIONS

**Shaoliang Zhu:** Investigation (equal); validation (equal). **Quan Cheng:** Investigation (equal); supervision (equal). **Mengjie Zou:** Investigation (equal); software (equal). **Chunxing Li:** Investigation (equal); software (equal). **Yi Tang:** Investigation (equal); validation (equal). **Longjie Xia:** Investigation (equal); software (equal). **Yanming Jiang:** Formal analysis (equal); software (equal). **Zheng Gong:** Methodology (equal); software (equal). **Zhenyong Tang:** Formal analysis (equal); validation (equal). **Yuntian Tang:** Investigation (equal); supervision (equal); writing – original draft (equal). **Honglin Luo:** Data curation (equal); visualization (equal). **Ningfu Peng:** Methodology (equal); supervision (equal). **Xiaojing Wang:** Methodology (equal); software (equal). **Xiaofeng Dong:** Project administration (equal); writing – original draft (equal).

## FUNDING INFORMATION

This study is supported by Guangxi Natural Science Fundation (2023JJA141310) to SLZ, Guangxi Natural Science Fundation (2023JJB140031) to MJZ, National Natural Science Foundation Project of China (82360495) to HLL and National Natural Science Foundation Project of China (82360488) to NFP.

## CONFLICT OF INTEREST STATEMENT

The authors declare that the research was conducted in the absence of any commercial or financial relationships that could be construed as a potential conflict of interest.

## Supporting information


Figure S1.

Figure S2.

Figure S3.

## Data Availability

The datasets of single‐cell sequencing generated and/or analysed during the current study are publicly available in the GEO. The original contributions presented in the study are included in the article/Supplementary Material. The inquiries of original contributions presented in the study can be directed to the corresponding authors.
