# Supplementary figures and images for: Combining bulk and scRNA‐seq to explore the molecular mechanisms governing the distinct efferocytosis activities of a macrophage subpopulation in PDAC
Source: J Cell Mol Med. 2024 Mar 19;28(7):e18266. doi: 10.1111/jcmm.18266 (PMC10949604; doi:10.1111/jcmm.18266)

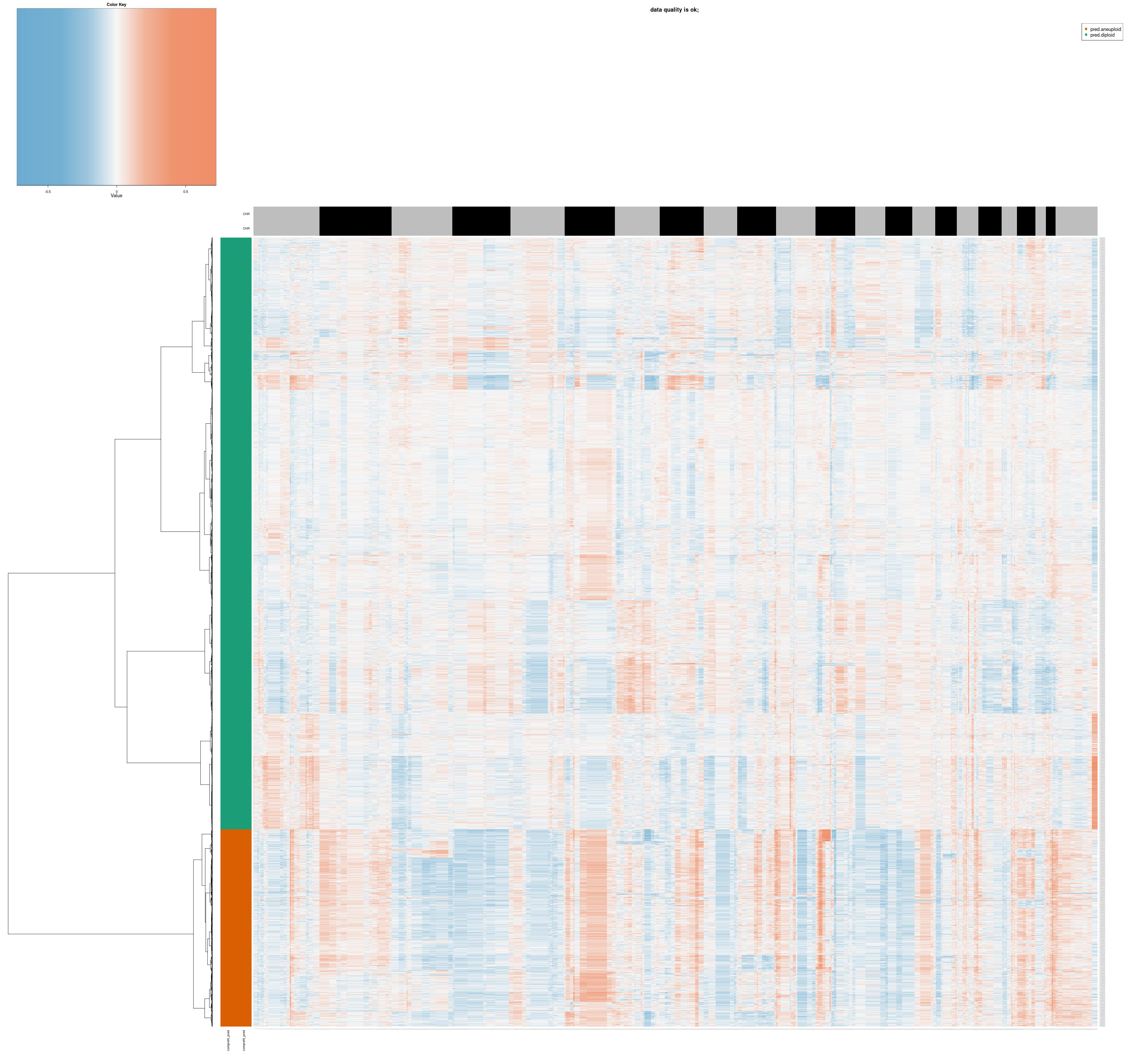

Supplement: Supplementary file 1 — Figure S1. [file JCMM-28-e18266-s003.tif]

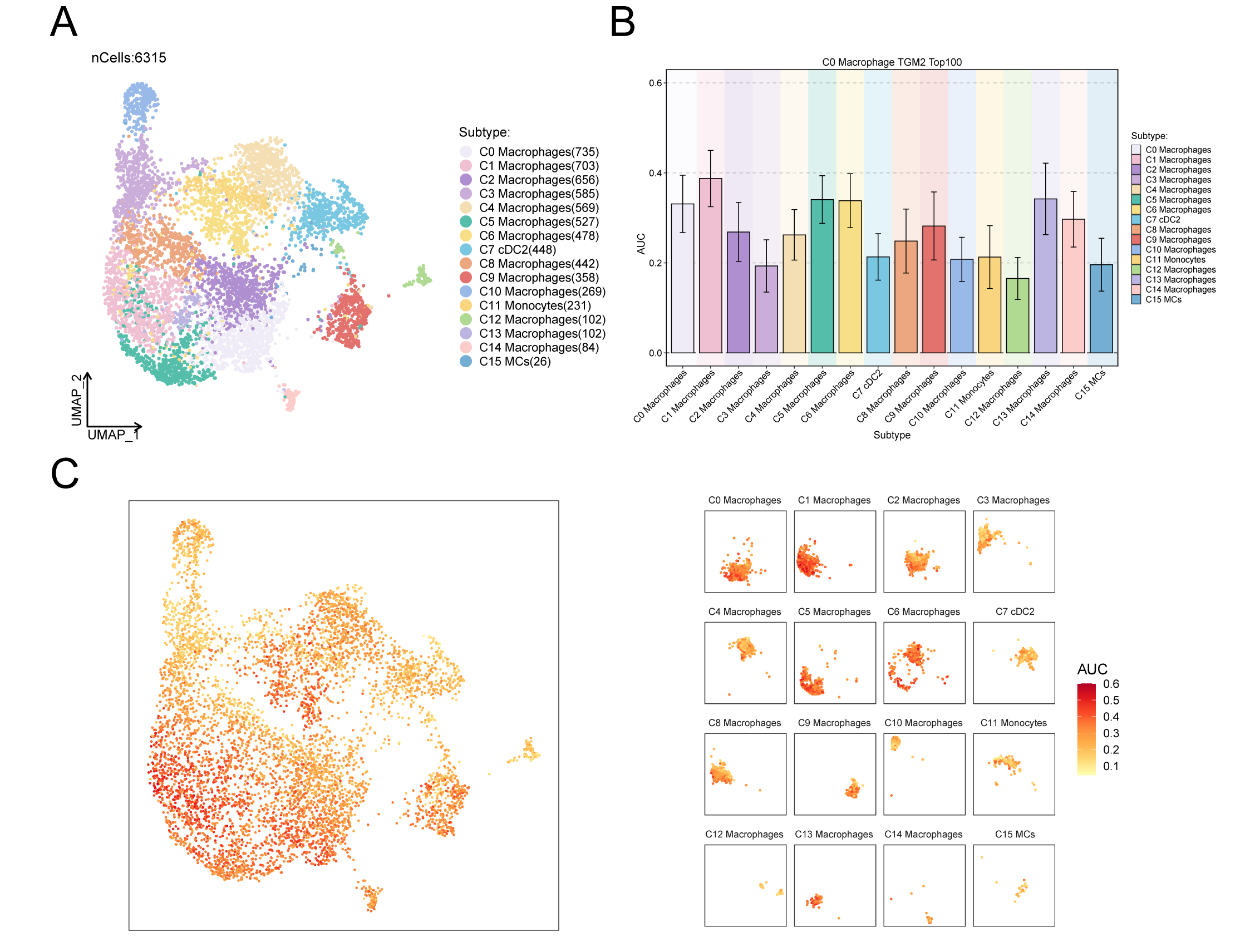

Supplement: Supplementary file 2 — Figure S2. [file JCMM-28-e18266-s002.tif]

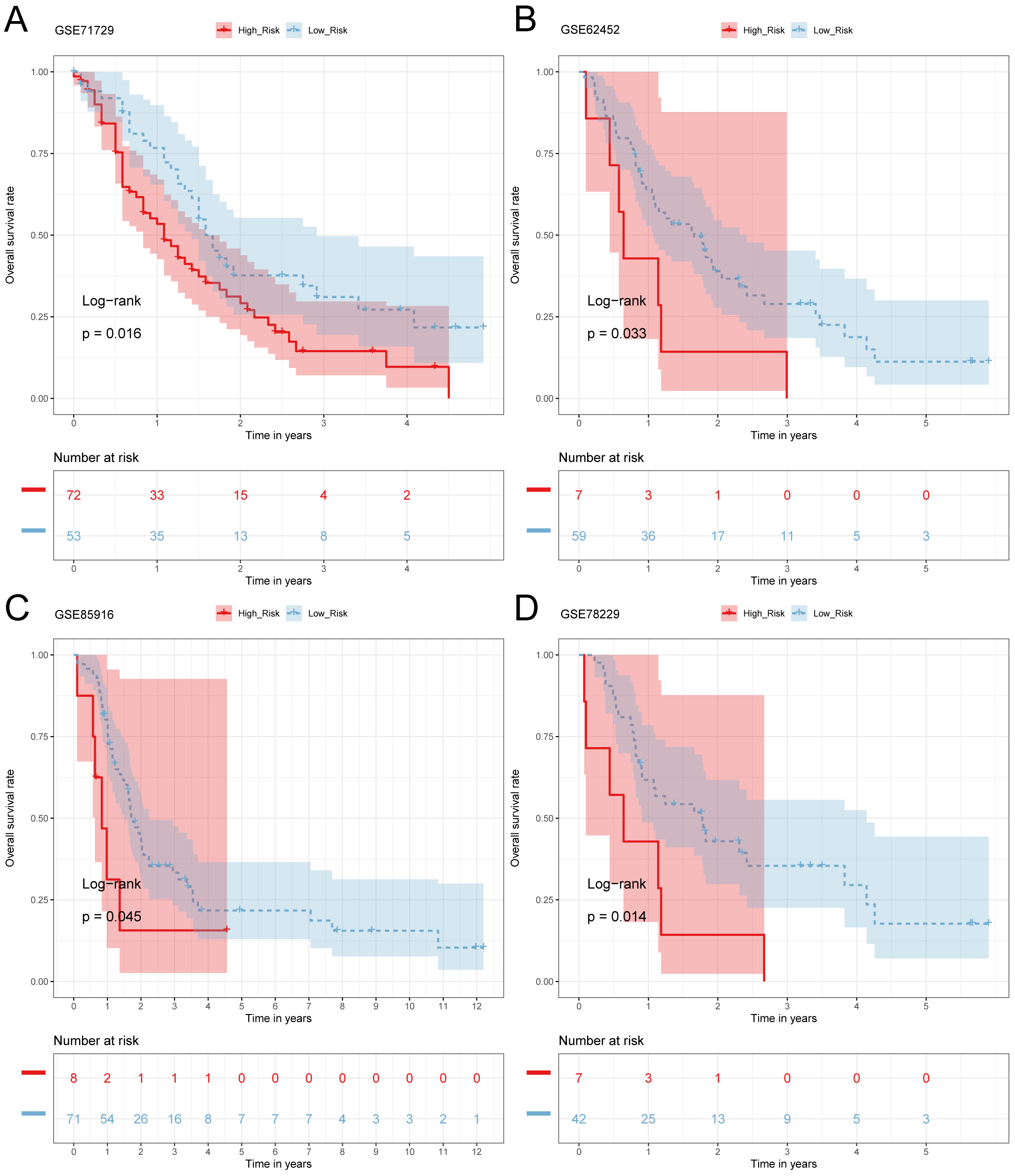

Supplement: Supplementary file 3 — Figure S3. [file JCMM-28-e18266-s001.tif]
